# Novel Mechanisms Modulating Palmitate-Induced Inflammatory Factors in Hypertrophied 3T3-L1 Adipocytes by AMPK

**DOI:** 10.1155/2018/9256482

**Published:** 2018-03-11

**Authors:** Naru Morita, Toshio Hosaka, Atsuko Kitahara, Toshitaka Murashima, Hirohisa Onuma, Yoshikazu Sumitani, Kazuto Takahashi, Toshiaki Tanaka, Takuma Kondo, Hitoshi Ishida

**Affiliations:** Third Department of Internal Medicine, Division of Diabetes, Endocrinology and Metabolism, Kyorin University School of Medicine, Tokyo, Japan

## Abstract

**Objective:**

A growing body of evidence indicates that AMP-activated protein kinase (AMPK) contributes to not only energy metabolic homeostasis but also the inhibition of inflammatory responses. However, the underlying mechanisms remain unclear. To elucidate the role of AMPK, in this study, we observed the effects of AMPK activation on monocyte chemoattractant protein-1 (MCP-1) release in mature 3T3-L1 adipocytes.

**Methods:**

We observed signal transduction pathways regulating MCP-1, which increased in obese adipocytes, in an *in vitro* model of hypertrophied 3T3-L1 adipocytes preloaded with palmitate.

**Results:**

Palmitate-preloaded cells exhibited significant increase in MCP-1 release and triglyceride (TG) deposition. Increased MCP-1 release and TG deposition were significantly decreased by an AMPK activator. In addition, the AMPK activator not only markedly diminished MCP-1 secretion but also augmented phosphorylation of nuclear factor-*κ*B (NF-*κ*B) and extracellular signal-regulated kinase (ERK) 1/2. In contrast, MCP-1 release suppression was abolished by the AMPK inhibitor compound C and the MEK inhibitor U0126.

**Conclusions:**

MCP-1 release from hypertrophied adipocytes is suppressed by AMPK activation through the NF-*κ*B and ERK pathways. These findings provide evidence that AMPK plays a crucial role in ameliorating obesity-induced inflammation.

## 1. Introduction

Chronic low-grade inflammation in adipose tissues of obesity models has been proven to play crucial roles in the development of obesity, which in turn induces systemic insulin resistance, the early step in the pathogenesis of type 2 diabetes mellitus (T2DM) [1–3]. Adipocytes have recently been recognized not only as energy storage cells, but also as having functions in endocrine signaling by producing and secreting a variety of pro-inflammatory adipocytokines, such as monocyte chemoattractant protein-1 (MCP-1), vascular endothelial growth factor (VEGF), tumor necrosis factor-*α* (TNF-*α*), and interleukin-6 (IL-6) [4, 5].

Among these, MCP-1 is one of the crucial adipocytokines, which accelerates macrophage infiltration into adipose tissue via the MCP-1 receptor, a CC chemokine receptor-2, and induces chronic low-grade inflammation [6, 7]. We previously showed that MCP-1 release is increased by activated c-Jun N-terminal kinase (JNK) and nuclear factor-*κ*B (NF-*κ*B) pathways in hypertrophied adipocytes [8]. Such increased levels of MCP-1 release recruit more macrophages to sites of infiltration, and these activated macrophages stimulate further production of MCP-1. Taking these observations together, the secretory system of MCP-1 can be understood as being critical for regulating inflammation in the adipose tissues of obesity models, which leads to exacerbation of obesity-related insulin resistance.

Adenosine monophosphate-activated protein kinase (AMPK) is a serine/threonine kinase which is highly conserved. It is referred to as a “metabolic master switch” based on its roles in regulating energy homeostasis and monitoring cellular energy stores by maintaining the balance between ATP production and consumption.

5-Aminoimidazole-4-carboxamide-1-*β*-d-ribofuranoside (AICAR), A769662, and metformin are well-known AMPK activators. However, these compounds have different mechanisms of action: AICAR is taken into cells by adenosine transporters and is converted into ZMP, which mimics the effect of AMP on AMPK activation in cells [9, 10], A769662 directly binds to an AMPK site [11], and metformin activates AMPK indirectly by changing the AMP/ATP ratio or through inhibition of mitochondrial respiratory chain complex I [12].

We previously reported that activation of endogenous AMPK by 2,4-dinitrophenol or AICAR significantly decreases the release of MCP-1 from mature 3T3-L1 adipocytes [13]. Several other studies have indicated that AMPK has a role in regulating inflammatory responses in various cells [14]. However, the molecular mechanism underlying this pathway is not fully understood. In this study, to elucidate the role of AMPK, we demonstrated the direct effects of AMPK using an *in vitro* model of artificially hypertrophied mature 3T3-L1 adipocytes preloaded with palmitate by applying the three aforementioned AMPK activators, AICAR, A769662, and metformin, focusing especially on the AMPK-mediated mechanisms regulating the expressions and secretions of adipokines playing central roles in the induction of peripheral insulin resistance.

## 2. Materials and Methods

### 2.1. Reagents

AICAR and palmitate were purchased from Wako (Osaka, Japan). A769662 and metformin hydrochloride were from Abcam (Cambridge, UK). Antibody against MCP-1 was obtained from R&D Systems (Minneapolis, MN, USA). Antibodies against AMPK*α*, phosphorylated AMPK*α*, p38 mitogen-activated protein kinase (MAPK), phosphorylated p38 MAPK, phosphorylated p44/42 MAPK (ERK1/2) (Thr202/Tyr204), phosphorylated acetyl-CoA carboxylase (ACC) (Ser79), ACC, NF-*κ*B p65, phosphorylated NF-*κ*B p65 (Ser536), phosphorylated JNK, and JNK were all obtained from Cell Signaling Technology (Danvers, MA, USA).

### 2.2. Preparation and Treatment of 3T3-L1 Adipocytes

The 3T3-L1 cells were obtained from the cell bank of the Japanese Collection of Research Bioresources (Tokyo, Japan). Cells were seeded and fed every two days in Dulbecco's modified Eagle's medium (DMEM) containing 25 mmol/L glucose supplemented with 50 U/mL penicillin, 50 *μ*g/mL streptomycin, 100 mmol/L minimum essential medium sodium pyruvate, and 10% fetal calf serum. Cells were grown under 5% CO_2_ at 37°C. Two days after the cells had reached confluence, differentiation was induced by addition of medium containing 500 *μ*mol/mL 3-isobutyl-1-methylxanthine (IBMX) (Wako), 100 nmol/L dexamethasone (Wako), and 1.7 *μ*mol/L insulin (Sigma). After 48 h, this mixture was replaced with fresh medium. The medium was then changed every two days until the cells were used for experiments. On day 10 after the induction of adipocyte differentiation, these differentiated 3T3-L1 adipocytes were treated with AICAR, A769662, metformin, or vehicle alone for one hour, then treated with palmitate. The concentrations of each reagent are given in Results or in the figure legends. At 12 hr or 24 hr after the addition of palmitate, several different analyses of the cells were conducted.

### 2.3. Immunoblotting

At 24 hr after the above treatments, cultured 3T3-L1 adipocytes were washed twice with ice-cold phosphate buffered saline (PBS), lysed in RIPA buffer (Nacalai Tesque, Kyoto, Japan) containing 50 mmol/L Tris-HCl buffer (pH 7.6), 150 mmol/L NaCl, 1% Nonidet P40, 0.5% sodium deoxycholate, and 1% protease inhibitor cocktail, with the addition of 0.1% sodium lauryl sulfate (SDS). Then, the cell lysates were sonicated and centrifuged for 10 min at 10000*g* at 4°C, and supernatants were collected. The 30 *μ*g of supernatant obtained was boiled in 1% SDS sample buffer in the presence of 50 mmol/L dithiothreitol. For the measurement of secreted proteins, cultured medium samples in the same amounts as those after the treatments were also used for immunoblotting. The samples were then subjected to SDS–polyacrylamide gel electrophoresis (SDS-PAGE) and transferred onto polyvinylidene difluoride membranes (GE Healthcare Little Chalfont, Buckinghamshire, England). Membranes were incubated with primary antibodies as described in Reagents and thereafter with horseradish peroxidase-conjugated secondary antibody. Protein bands were visualized with Chemi-Lumi One Super reagents according to the manufacturer's protocol (Nacalai Tesque), followed by X-ray film exposure. Images and densitometry were obtained with ImageQuant LAS 4000 version 1.2 and ImageQuant TL 7.0 (GE Healthcare Little Chalfont). Protein band intensities under basal conditions were set as 100% for normalization purposes.

### 2.4. Quantitative Real-Time RT-PCR

Total RNA was extracted from 3T3-L1 adipocytes using the RNAqueous®-4PCR kit (Ambion, Austin, TX, USA) according to the manufacturer's instructions at 12 hr after the palmitate treatment. Quantitative real-time RT-PCR was conducted using the 7300 real-time PCR system (Applied Biosystems, Foster City, CA, USA). MCP-1 (Mm00441242_m1) primer and probe was ordered from Applied Biosystems. The mRNA signal was normalized over the 18S rRNA signal. The mean value of each experiment, performed in triplicate, was used to determine the relative mRNA level.

### 2.5. Enzyme-Linked Immunosorbent Assay (ELISA)

Twenty-four hours after palmitate treatment, the culture medium was collected and MCP-1 secretion was measured employing a CCL2/MCP-1 ELISA kit (R&D Systems). The protein concentration was calculated from the standard curve and adjusted by the intracellular protein contents. The protein concentration under basal conditions was set to 100% for normalization purposes.

### 2.6. Analysis of Triglycerides (TG)

At 24 hr after treatment, intracellular TG levels in 3T3-L1 adipocytes were determined employing a commercially available Lipid Assay kit (Cosmo Bio, Tokyo, Japan) [15], according to the manufacturer's protocol, and the values obtained were adjusted to the intracellular total protein contents. For Lipid Assay kit, cells were fixed overnight at room temperature with 10% formalin neutral buffer solution (Wako) and then stained with oil red O solution for 15 min. Oil red O solution was then removed, and the cells were washed. After drying, the cells were observed with a KEYENCE BZ-X700 All-in-one Fluorescence Microscope and extraction reagent was added to measure dye extraction (540 nm) with a plate reader, to allow calculation of the TG contents.

### 2.7. Statistical Analysis

Statistical analyses were performed employing the *unpaired t-test* or *analysis of variance* (*ANOVA*) and post hoc analysis using Bonferroni's method. Results were expressed as the means ± SEM, and differences at a value of *p* < 0.05 were considered to be statistically significant.

## 3. Results

### 3.1. Effects of AICAR on MCP-1 Expression and Its Release from Hypertrophied 3T3-L1 Adipocytes

**Figure 1:**
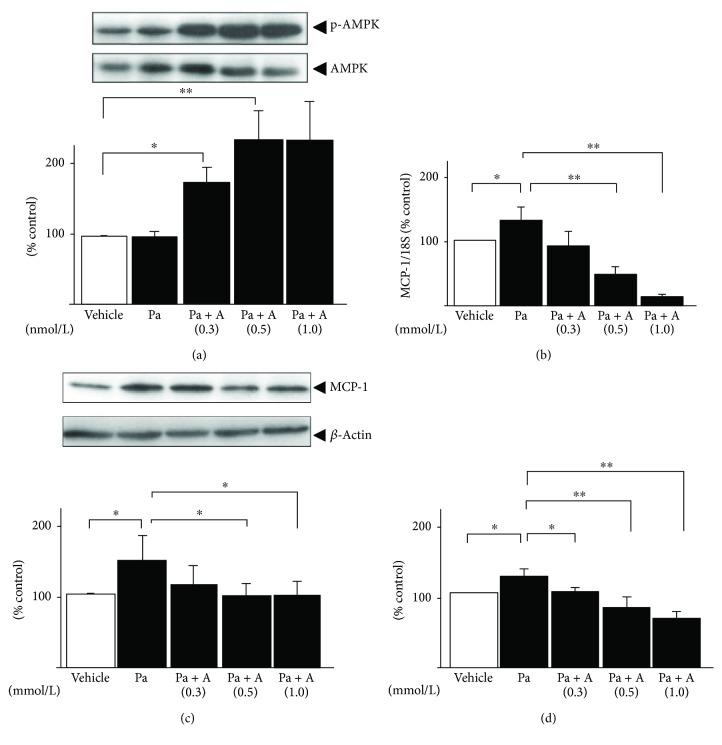
Effects of AICAR on the mRNA expressions and release of MCP-1 in 24 h palmitate-preloaded 3T3-L1 adipocytes. Differentiated 3T3-L1 adipocytes were preincubated with 0.3–1.0 mmol/L of AICAR (a) for 1 h and then treated with 0.3 mmol/L palmitate (Pa) (black bar) or ethanol vehicle alone (white bar) for 24 h. AMPK phosphorylation of Thr172 (a) and intracellular MCP-1 (c) was quantified by immunoblot analysis, and MCP-1 release was also assessed by ELISA (d). The mRNA levels of MCP-1 (b) were measured by quantitative real-time RT-PCR at 12 h after stimulation and then normalized over the 18S rRNA signal. Data are means ± SEM (*n* = 4). ^∗^*p* < 0.05, ^∗∗^*p* < 0.01 compared to corresponding control cells.

We initially monitored the effects of AMPK activation on palmitate-induced MCP-1 expression. In 3T3-L1 adipocytes, AICAR (0.3–1.0 mmol/L) pretreatment 24 h prior to the addition of palmitate dose-dependently increased the phosphorylation of AMPK (Figure 1(a)). Meanwhile, the palmitate-stimulated elevation of MCP-1 expression was decreased by pretreatment of AICAR dose-dependently (Figure 1(b)). AICAR at a dose of 0.5 mmol/L, which blunted the enhancement of MCP-1 mRNA by 64% (Figure 1(b), *p* < 0.01), intracellular MCP-1 protein by 34% (Figure 1(c), *p* < 0.05), and MCP-1 release by 35% (Figure 1(d), *p* < 0.05), was the minimum concentration of AICAR, which reduced MCP-1 expression overall, and thus, we used this dose for all further observations.

### 3.2. Signaling Mechanisms Involved in Palmitate-Induced MCP-1 Release and the Inhibitory Role of AICAR

**Figure 2:**
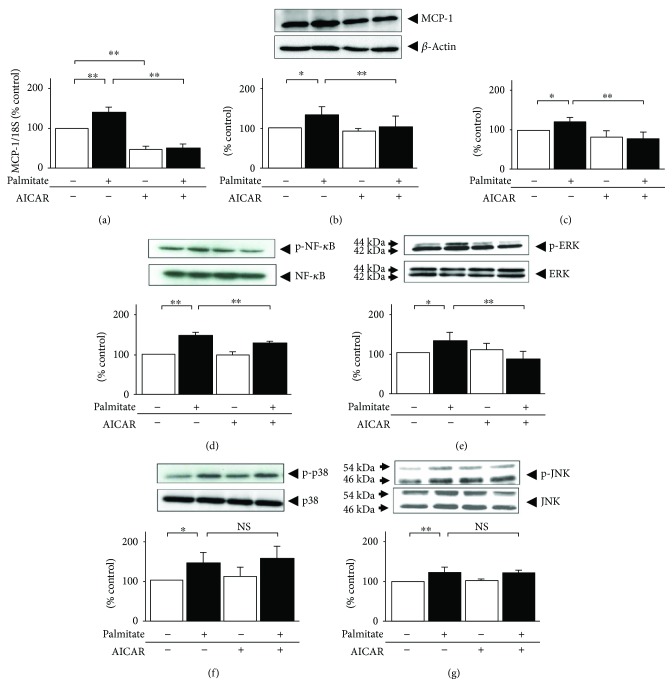
Effects of AICAR on palmitate-induced MCP-1, NF-*κ*B, and MAPK signaling in 3T3-L1 adipocytes. 3T3-L1 cells were exposed to 0.3 mmol/L palmitate (black bar) or ethanol vehicle alone (white bar) in the presence or absence of 0.5 mmol/L AICAR for 12 h. The mRNA levels of MCP-1 were measured by quantitative real-time RT-PCR (a). After additional 12 h, cell lysates were immunoblotted to determine the intracellular MCP-1 concentration (b), phosphorylation of NF-*κ*B on Ser536 (d), ERK1/2 on Thr180/Tyr182 (e), p38 MAPK on Thr180/Tyr182 (f), and JNK on Thr183/Tyr185 (g). *β*-Actin was measured as an internal control, and each phosphorylation was normalized by the corresponding total protein concentration. The release of MCP-1 was also assessed by ELISA (c). Data are means ± SEM (*n* = 4). ^∗^*p* < 0.05, ^∗∗^*p* < 0.01 compared to corresponding control cells. NS: no significant difference compared to corresponding control cells.

To elucidate the mechanism by which AICAR prevents the palmitate-stimulated MCP-1 secretion cascade (Figures 2(a)–2(c)), we first examined the involvement of NF-*κ*B signaling, which has been characterized as an activator of the expressions of many genes and is considered to be crucial in obesity-induced inflammatory signaling [8, 16]. As shown in Figure 2(d), palmitate-induced NF-*κ*B phosphorylation in 3T3-L1 adipocytes and AICAR antagonized this palmitate-induced activation of NF-*κ*B. Since the NF-*κ*B pathway serves as a target for MAPKs [16], we next investigated the involvement of subfamilies of MAPKs: ERK, p38 MAPK, and JNK. All members of the MAPK family were significantly activated after 24 h of palmitate treatment: ERK increased 1.3-fold, p38 MAPK increased 1.4-fold, and JNK increased 1.2-fold (*p* < 0.05, *p* < 0.05, and *p* < 0.01; Figures 2(e)–2(g), resp.). On the other hand, AICAR pretreatment significantly suppressed the increment in ERK phosphorylation, that is, by 42% (*p* < 0.01), while no effect was observed on the palmitate-induced increases in JNK and p38 MAPK phosphorylation.

### 3.3. Effects of AICAR on Intracellular TG Contents in Palmitate-Preloaded 3T3-L1 Adipocytes

**Figure 3:**
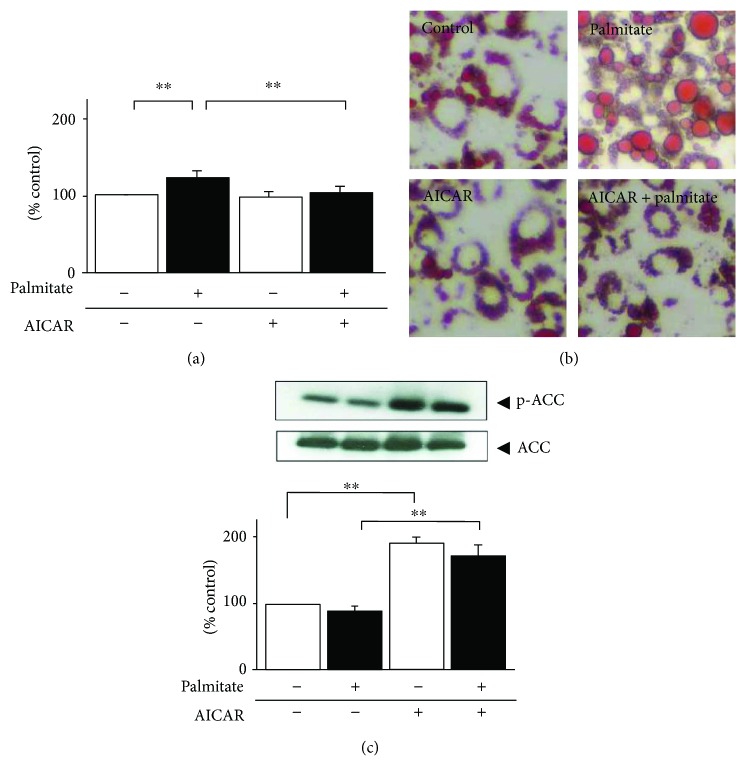
Effects of AICAR on intracellular TG contents in 24 h palmitate-preloaded 3T3-L1 adipocytes. Differentiated 3T3-L1 adipocytes were exposed to 0.3 mmol/L palmitate (black bar) or ethanol vehicle alone (white bar) in the presence or absence of 0.5 mmol/L AICAR for 24 h. (a) Cellular TG contents were measured, and the concentrations were then adjusted to intracellular total protein contents. (b) Lipid drops were stained with oil red O and examined using fluorescence microscope. (c) ACC phosphorylation on Ser79 was then quantified by immunoblot analysis. Data are means ± SEM (*n* = 4). ^∗∗^*p* < 0.01 compared to corresponding control cells.

Palmitate exacerbates adipocyte hypertrophy via TG deposition, as shown in Figure 3(a) (1.2-fold, *p* < 0.01) and Figure 3(b). Conversely, in response to pretreatment with AICAR which stimulated ACC phosphorylation (Figure 3(c)), the intracellular TG level decreased to 85% of that with palmitate alone (Figure 3(a), *p* < 0.01).

### 3.4. Effects of A769662 on MCP-1 Expression and Its Release from Hypertrophied 3T3-L1 Adipocytes and Intracellular TG Contents

**Figure 4:**
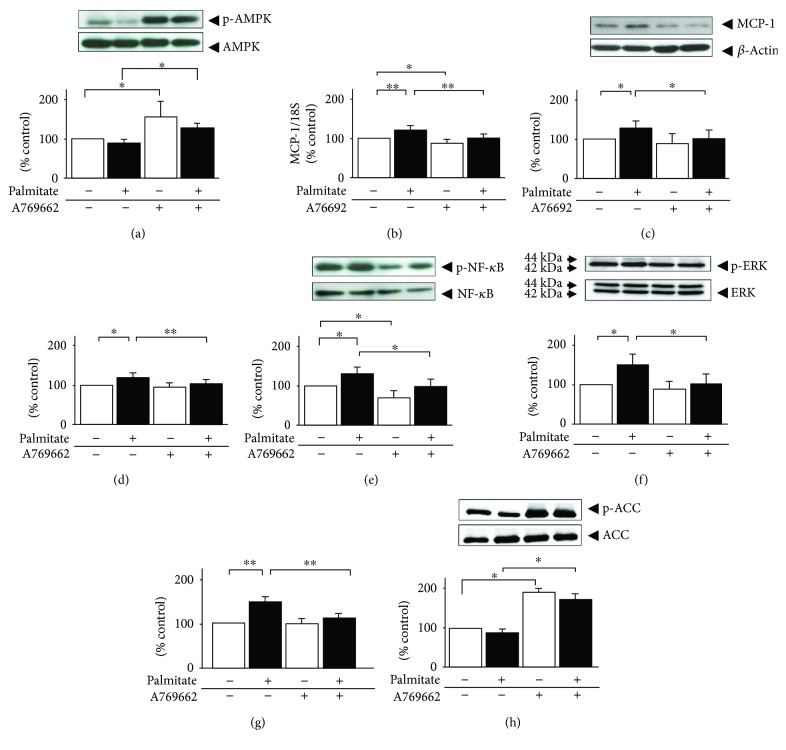
Effects of A769662 on MCP-1, NF-*κ*B, and MAPK signaling and intracellular TG contents in 24 h palmitate-preloaded 3T3-L1 adipocytes. Differentiated 3T3-L1 adipocytes were exposed to 0.3 mmol/L palmitate (black bar) or ethanol vehicle alone (white bar) in the presence or absence of 25 *μ*mol/L A769662 for 24 h. Lysates were immunoblotted to assess the phosphorylation of AMPK on Thr172 (a), NF-*κ*B on Ser536 (e), ERK1/2 on Thr180/Tyr182 (f), ACC on Ser79 (h), and intracellular MCP-1 (c). The release of MCP-1 was also assessed by ELISA (d). The cellular TG (g) contents were measured and then adjusted to intracellular total protein contents. The levels of MCP-1 mRNA (b) were measured by quantitative real-time RT-PCR at 12 h after stimulation and then normalized over the 18S rRNA signal. Data are means ± SEM (*n* = 4). ^∗^*p* < 0.05, ^∗∗^*p* < 0.01 compared to corresponding control cells.

ZMP, an AICAR metabolite, has been found to regulate other AMP-sensitive enzymes [10]. In contrast, A769662 reportedly does not exert this effect [11]. To verify the effects of AMPK on palmitate-preloaded adipocytes, we next examined whether A769662, a specific AMPK activator, also antagonizes the pro-inflammatory effect of palmitate. As expected, the A769662 treatment significantly increased, by 1.6-fold, the phosphorylation of AMPK (Figure 4(a), *p* < 0.05). Treatment with 25 *μ*mol/L A769662 plus palmitate significantly inhibited MCP-1 mRNA, intracellular MCP-1 protein, MCP-1 release, and activation of NF-*κ*B (Figures 4(b)–4(e)), by 28% (*p* < 0.01), 20% (*p* < 0.05), 14% (*p* < 0.05), and 25% (*p* < 0.05), respectively. ERK phosphorylation was also inhibited by treatment with A769662, by 31% (Figure 4(f), *p* < 0.05). In addition, A769662 pretreatment increased the phosphorylation of ACC and resulted in the inhibition of intracellular TG accumulation (Figures 4(g) and 4(h)). However, much like AICAR, A769662 did not antagonize palmitate-induced phosphorylation of JNK and p38 MAPK (data not shown).

### 3.5. Effects of Metformin on MCP-1 Expression and Its Release from Hypertrophied 3T3-L1 Adipocytes and Intracellular TG Contents

**Figure 5:**
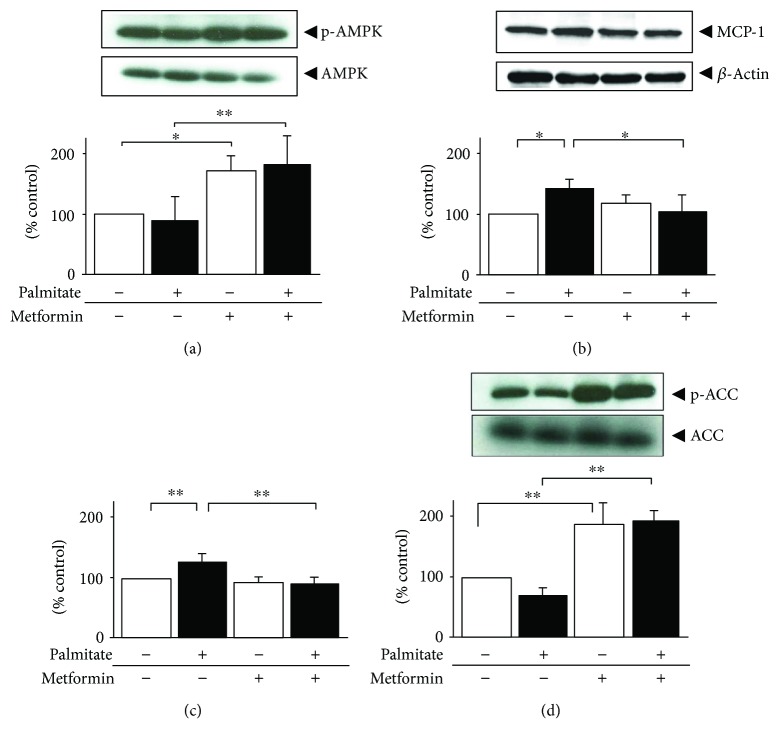
Effects of metformin on palmitate-induced MCP-1 protein and intracellular TG contents in 24 h palmitate-preloaded 3T3-L1 adipocytes. Differentiated 3T3-L1 adipocytes were exposed to 0.3 mmol/L palmitate (black bar) or ethanol vehicle alone (white bar) in the presence or absence of 2.5 mmol/L metformin for 24 h. Lysates were immunoblotted to assess the phosphorylation of AMPK on Thr172 (a) and intracellular MCP-1 (b). The cellular TG (c) contents were measured and then adjusted to intracellular total protein contents. Data are means ± SEM (*n* = 4). ^∗^*p* < 0.05, ^∗∗^*p* < 0.01 compared to corresponding control cells.

Metformin is an established first-line therapy for T2DM. One of the important targets of metformin is AMPK. In a past study, metformin was shown to significantly improve MCP-1 levels in the aorta [17], and this drug also reportedly inhibits NF-*κ*B activation via AMPK activation in vascular endothelial cells [18]. Because the AMPK activators, AICAR and A769662, used in our present study moderated the pro-inflammatory potential of palmitate, we next analyzed whether treatment with metformin might affect palmitate-stimulated adipokine secretion from hypertrophied 3T3-L1 adipocytes. Metformin 2.5 mmol/L treatment resulted in a 1.7-fold increase in the phosphorylation of AMPK (Figure 5(a), *p* < 0.05). Palmitate-induced intracellular MCP-1 protein was significantly inhibited by treatment with metformin (−28%; Figure 5(b), *p* < 0.05). Metformin also significantly attenuated the palmitate-induced increases in MCP-1 mRNA, MCP-1 release (−21%, −53%, resp.), NF-*κ*B, and ERK activation (data not shown). Inhibition of intracellular TG accumulation was also observed with a concomitant increase in the phosphorylation of ACC (Figures 5(c) and 5(d)).

### 3.6. Signaling Mechanism Involved in AMPK Inhibition of Palmitate-Induced MCP-1 Expression in Hypertrophied 3T3-L1 Adipocytes

**Figure 6:**
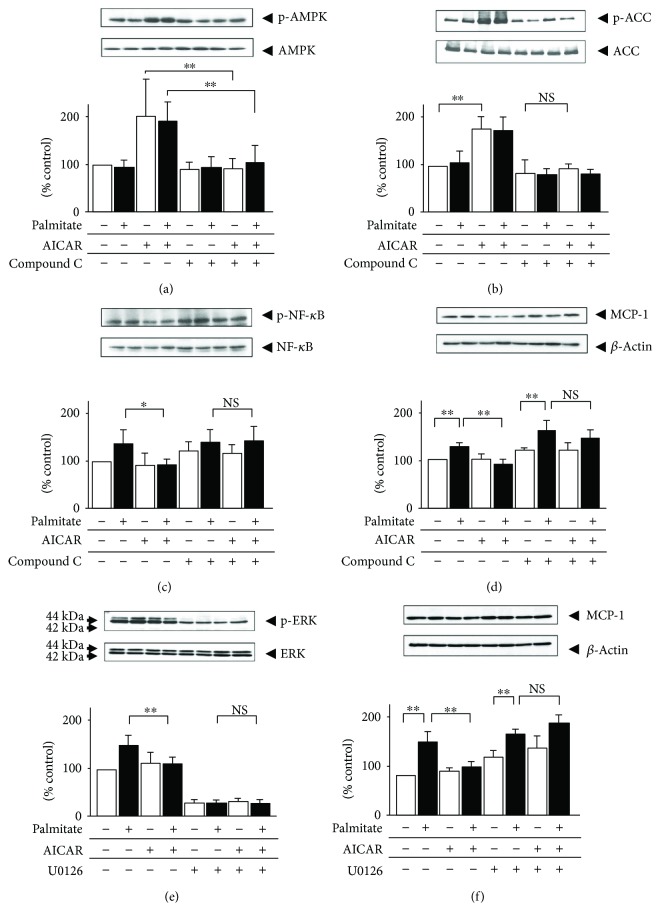
Effects of compound C and U126 on MCP-1 in palmitate-preloaded 3T3-L1 adipocytes treated with AICAR. Adipocytes were pretreated with 10 *μ*mol/L compound C (a, b, c, and d), 10 *μ*mol/L U126 (e, f), or vehicle (dimethyl sulfoxide) alone for 20 min. Then, the cells were treated with 0.3 mmol/L palmitate (black bar) or vehicle (ethanol) alone (white bar) for 24 h with or without 0.5 mmol/L AICAR. Intracellular MCP-1 (d, f) was quantified by immunoblotting. AMPK phosphorylation on Thr172 (a), ACC phosphorylation on Ser79 (b), NF-*κ*B phosphorylation on Ser536 (c), and ERK1/2 phosphorylation on Thr180/Tyr182 (e) were also quantified by immunoblot analysis. Each phosphorylation was normalized by the level of the corresponding total protein. *β*-actin was assessed as an internal control. Results are means ± SEM (*n* = 3). ^∗^*p* < 0.05, ^∗∗^*p* < 0.01 compared to the corresponding controls. NS: no significant difference compared to corresponding control cells.

Given that AMPK activation blocked intracellular MCP-1 protein production and regulated this palmitate-induced NF-*κ*B signaling in a solely ERK-dependent manner, as shown in Figure 2, we assessed whether the phosphorylation of NF-*κ*B and ERK1/2 is directly downstream from AMPK. Treatment with the AMPK inhibitor compound C (10 *μ*mol/L) clearly reduced the phosphorylation induced by AMPK (Figure 6(a)). Blockage of AMPK abolished the AICAR-mediated increase in ACC phosphorylation and decrease in both NF-*κ*B phosphorylation and intracellular MCP-1 protein (Figures 6(b)–6(d)). In addition, after treatment with 10 *μ*mol/L of the MEK inhibitor U0126, the AICAR-mediated decrease in the expression of intracellular MCP-1 was also abolished (Figures 6(e) and 6(f)).

## 4. Discussion

There is a growing evidence that increased inflammation in tissues is a key characteristic of obesity and contributes to malfunctions of tissues and organs [19–21]. Excessive saturated fatty acids, such as palmitate, have been shown to enhance the pro-inflammatory state in adipocytes, skeletal muscle, pancreatic *β* cell lines, and osteoblasts [8, 22–25]. However, the mechanisms by which saturated fatty acids promote the inflammatory state have not been fully elucidated. On the other hand, extensive studies have indicated that AMPK activation may have anti-inflammatory effects [26, 27]. Moreover, it was reported that AMPK activation increases fatty acid oxidation and inhibits lipogenesis through phosphorylation of ACC, which results in decreased lipid deposition in liver and muscle [28].

In this study, we employed an artificial hypertrophied adipocyte model with palmitate preloading and compared the effect of AMPK activation on intracellular signal transduction pathways involved in MCP-1 release and lipid deposition. We previously confirmed that treatment with AICAR inhibited MCP-1 release [13] and that MCP-1 release is potentially enhanced via NF-*κ*B pathways in hypertrophied adipocytes [8]. Therefore, we attempted to analyze the expression and secretion of MCP-1 and NF-*κ*B activation. MCP-1 expression/secretion and NF-*κ*B activation were significantly increased by palmitate stimulation and were inhibited by individual treatments with all of the three AMPK activators, AICAR, A769662, and metformin, despite these AMPK activations having different mechanisms. Also in the previous study [29], we have demonstrated that in hypertrophied adipocytes, the expression and secretion of VEGF_120_ increases through PI3K; therefore, we also analyzed whether AMPK exertion has a protective effect against palmitate-induced VEGF_120_ and activation of Akt. However, AMPK activation exhibited no effects on Akt phosphorylation or on the expression and secretion of VEGF_120_ (data not shown). Thus, we focused on MCP-1 secretion and conducted further studies to elucidate the intracellular signaling pathways related to MCP-1-NF-*κ*B cascades. Palmitate stimulation markedly enhanced the phosphorylation of JNK, p38, and ERK1/2 in 3T3-L1 adipocytes, whereas among the members of the MAPK family, only ERK1/2 phosphorylation was suppressed. Compound C is a competitive inhibitor binding to the same ligand site as AMP or AICAR, such that it can block AICAR-induced AMPK activation [30]. U0126 is a MEK1/2 inhibitor, and it has been widely used to elucidate the functions of ERK1/2, as ERK1/2 is one of the downstream targets of MEK1/2 [31]. To understand the association of the AMPK system with AMPK activator treatment, we next investigated the effects of compound C and U126 on intracellular MCP-1 expression. Treatment with compound C did not significantly affect the augmented intracellular MCP-1 protein nor the NF-*κ*B activity induced by palmitate, while treatment with compound C inhibited AMPK activator-induced ACC phosphorylation (Figure 6). Similarly, treatment with U0126 did not reduce palmitate-induced intracellular MCP-1 protein (Figure 6). Taken together, these observations suggest that AMPK reduces the pro-inflammatory state by inhibiting MCP-1 expression via NF-*κ*B-ERK1/2 pathways and by reducing lipid deposition via phosphorylation of ACC in hypertrophied adipocytes.

Dai et al. reported that AICAR and metformin downregulated palmitate-induced MAPK activation via different mechanisms in a *β* cell line: AICAR by reversing TG overload, activating Akt, and inhibiting p38 MAPK, whereas metformin through suppression of JNK and p38 MAPK [25]. Sena et al. showed that metformin treatment improved not only the CCL2 level but also oxidative stress in the aortas of high-fat-fed diabetic rats [17]. However, in our study, none of the AMPK activators suppressed neither JNK nor p38 phosphorylation. This might be explained by the differences in cells lines. In osteoblasts, palmitate-induced apoptosis was inhibited by AICAR via ERK activation [24]. In rat skeletal muscle cells, ERK played a key role in palmitate-induced activation of NF-*κ*B signaling and AMPK blunted this inflammatory pathway [23]. AICAR suppressed TNF-*α*-induced phosphorylation of ERK [32] in adipose tissue of db/db mice and in 3T3-L1 adipocytes. Because adipocytes and osteogenic cells share a common precursor in adult marrow, there is a high degree of plasticity between the two cell lines even at the most advanced stages of maturation [33–35]. Moreover, skeletal myocytes originate from precursors in the somite that also give rise to adipocytes. These previous studies support our hypothesis that AMPK activation inhibits palmitate-induced MCP-1 via NF-*κ*B-ERK-dependent pathways. We cannot exclude the possibility that MAPK upregulation or blockage of lipid deposition by AMPK activator prevents fatty acid-induced insulin resistance by directly targeting proximal components of the insulin signaling cascade [36]. Nor were we able to determine whether the decrease in NF-*κ*B is influenced only by ERK1/2. However, Green et al. proved that ERK plays a key role in palmitate-induced IKK/NF-*κ*B activation by using PMA, a potent ERK activator, and that AMPK activators blocked the effects of palmitate by reducing ERK signaling [23].

## 5. Conclusions

In this study, we showed that not only AICAR, but also A769662, a specific AMPK activator, and metformin, which is known as the first-line therapy for T2DM, antagonized the palmitate-induced ERK-NF-*κ*B activation concomitant with the MCP-1 reduction. These results clearly indicate AMPK activation to exert anti-inflammatory effects on saturated fat-treated 3T3-L1 adipocytes. Elucidating the details of the mechanisms underlying reduced MCP-1 expression in response to AMPK activation in hypertrophied 3T3-L1 adipocytes may open the way to new therapeutic strategies for obesity-induced inflammation and insulin resistance.
